# Shape Parameterization and Efficient Optimization Design Method for the Ray-like Underwater Gliders

**DOI:** 10.3390/biomimetics11010058

**Published:** 2026-01-08

**Authors:** Daiyu Zhang, Daxing Zeng, Heng Zhou, Chaoming Bao, Qian Liu

**Affiliations:** 1 School of Naval Architecture and Engineering, Jiangsu University of Science and Technology, Zhenjiang 212003, China; daiyu.zhang@just.edu.cn (D.Z.); daxing.zeng@stu.just.edu.cn (D.Z.); bao.chaoming@just.edu.cn (C.B.); liuqian@just.edu.cn (Q.L.); 2 China Ship Scientific Research Center, Wuxi 214000, China; 3 College of Shipbuilding Engineering, Harbin Engineering University, Harbin 150006, China

**Keywords:** ray-like underwater glider, parameterized modeling, hydrodynamic calculation, kriging surrogate model

## Abstract

To address the challenges of high computational cost and lengthy design cycles in the high-precision optimization of ray-like underwater gliders, this study proposes a high-accuracy, low-cost parametric modeling and optimization method. The proposed framework begins by extracting the characteristic contours of the manta ray and reconstructing the airfoil sections using the Class-Shape Transformation (CST) method, resulting in a flexible parametric geometry capable of smooth deformation. High-fidelity Computational Fluid Dynamics (CFD) simulations are employed to evaluate the hydrodynamic characteristics, and detailed flow field analyses are conducted to identify the most influential geometric features affecting lift and drag performance. On this basis, a Kriging-based sequential optimization framework is developed. The surrogate model is adaptively refined through dynamic infilling of sample points based on combined Mean Squared Prediction (MSP) and Expected Improvement (EI) criteria, thus improving optimization efficiency while maintaining predictive accuracy. Comparative case studies demonstrate that the proposed method achieves a 116% improvement in lift-to-drag ratio and a more uniform flow distribution, confirming its effectiveness in enhancing both design accuracy and computational efficiency. The results indicate that this approach provides a practical and efficient tool for the parametric design and hydrodynamic optimization of bio-inspired underwater vehicles.

## 1. Introduction

As terrestrial resource development approaches its limits, the global process of ocean exploration is accelerating. Against this backdrop, the innovation of ocean observation technology systems has become a key focus of strategic plans for various countries. As a typical representative of new marine detection equipment, the Autonomous Underwater Glider (AUG) [1,2,3], with its unique characteristics of low power consumption and long endurance, has demonstrated irreplaceable value in the field of deep-sea environmental monitoring. These autonomous navigation devices optimize energy utilization efficiency, achieving sustained operational capabilities that traditional observation equipment cannot match. Their modular design significantly reduces operation and maintenance costs. Currently, underwater gliders are widely used in fields such as ocean dynamic analysis, ecological element monitoring, and climate change research [4]. They have become one of the core components in building intelligent ocean observation networks, providing crucial technological support for human understanding and utilization of the ocean.

The underwater glider primarily converts vertical motion into horizontal forward propulsion through the coordination of hydrofoils and buoyancy control devices. Its overall performance and endurance are directly constrained by its lift–drag ratio characteristics. The flat-winged body configuration of ray-like organisms, which has evolved through long-term natural selection [5,6,7], naturally exhibits a high lift–drag ratio. This provides a biomimetic insight to overcome the performance limits of traditional combinations of rotary bodies and wings [8]. The ray-like underwater glider (RLUG), developed based on this biological prototype, significantly extends the gliding distance and operational range by reconstructing the fluid dynamic layout. However, the initial bio-inspired configuration still has room for improvement in its fluid dynamic characteristics. The lack of fine design in the shape may lead to issues such as local flow separation and vortex-induced drag, which in turn limit the full potential of the lift–drag ratio performance. Therefore, fluid dynamic optimization design method for the ray-like shape has become a key technological method for enhancing its hydrodynamic efficiency. By systematically improving the configuration parameters, the lift–drag ratio potential of the biomimetic design can be further liberated.

The shape optimization design system of the ray-like underwater glider includes three core components: shape parameterization modeling, high-precision hydrodynamic performance evaluation, and optimization strategy formulation. The optimization strategy serves as the top-level framework, coordinating the overall design process and guiding the optimization direction. Shape parameterization modeling establishes a mapping relationship between geometric features and design variables by constructing a set of geometric parameters. This transforms the complex geometric optimization problem into a mathematical optimization process within the parameter space. High-precision hydrodynamic performance evaluation is based on fluid dynamics principles, providing a quantitative analysis of the force characteristics and energy conversion efficiency of the design scheme in a specific flow field. These three components form a closed-loop iterative mechanism, collectively supporting the extraction of high lift–drag ratio characteristics and the improvement of fluid dynamic performance for the biomimetic configuration.

Computational Fluid Dynamics (CFD) [9,10,11] is a crucial tool for high-precision calculation of the hydrodynamic performance of underwater gliders. In the shape optimization process of underwater gliders, while CFD methods can provide highly accurate hydrodynamic performance evaluations, their computational resource consumption is extremely high, making them a major hindrance in the optimization process. Each adjustment of shape parameters requires the regeneration of the mesh and a new simulation calculation. This issue becomes even more serious when the design variables are high-dimensional, and the parameter space is vast, requiring extensive performance evaluations for a large number of sample points, which significantly increases the computational burden. Therefore, CFD methods not only severely slow down the optimization convergence of the ray-like underwater glider but also limit the comprehensive exploration of the global design space. There is an urgent need for more efficient alternative methods to enhance overall design efficiency.

Parameterized modeling methods allow designers to represent complex 3D shapes using simple mathematical parameters, enabling precise control over the shape [12,13,14]. Traditional modeling methods that rely on CAD tools are inefficient and struggle to support large-scale design space exploration. Additionally, designers cannot intuitively modify the shape based on physical meanings, particularly when defining the geometric constraints necessary for optimization problems [15]. Typical examples include Wang Kangjun et al. [16], who parameterized the shape of a wing-body fusion aircraft, which achieved multi-scale parametric control over the aircraft surface; Zhang Daiyu et al. [17], who also used FFD and axial deformation methods for parameterizing the shape of a wing-body integrated underwater glider. However, for the specific and constrained scenario of ray-like underwater glider shape parametrization, conventional parametric methods still do not offer a satisfactory solution.

Metaheuristic Optimization Algorithms methods (such as NSGA, PSO, BESO [18,19,20]) are widely used due to their strong global search capability and high adaptability, employing heuristic search to approach the global optimum. Typical applications include Jia et al. [21], who employed the NGSA-III algorithm to search for the optimal framework of a structure across the entire design space, achieving structural optimization for a robotic gripper unloading device; Shivendra Singh et al. [22] utilized the NSGA-II algorithm to efficiently optimize airfoil performance under low Reynolds number conditions. However, the convergence of these methods relies on a large number of iterations, making them computationally expensive and better suited for optimization problems with a smaller number of variables. Therefore, they are not applicable to the scenario in this study, which involves a ray-like underwater glider with a high-dimensional shape optimization problem.

To fulfill the high-precision design requirements of ray-like underwater gliders, a shape parameterization and efficient optimization approach is presented for a ray-like underwater glider. The specific research approach is as follows: First, the overall contour features of the ray biological prototype are systematically extracted and analyzed, and key morphological indicators are quantified through geometric parameterization techniques. Additionally, the CST method is introduced to model the airfoil profiles with high precision, establishing a mapping relationship between geometric parameters and shape features to achieve flexible control and deformation of the ray-like shape. Next, CFD methods are used to perform numerical calculations of hydrodynamic performance, obtaining key indicators such as lift, drag, and pressure distribution, which provide accurate performance evaluation criteria for subsequent optimization. Then, a sequential optimization framework based on a Kriging surrogate model is built. Through dynamic point addition strategies and the iterative updating mechanism of the Kriging model [23,24], the optimization accuracy is ensured while significantly improving computational efficiency, overcoming the efficiency bottleneck of traditional optimization methods in cases with high variable dimensions and complex constraints. Finally, through the optimization validation of the ray-like underwater glider shape, the feasibility and effectiveness of the method built before to improve design efficiency and optimize hydrodynamic performance are validated, providing a new technical pathway for fluid dynamic design of biomimetic underwater equipment.

The remainder of this paper is organized as follows: Section 2 presents the geometric parameterization method and shape modeling strategy of the ray-like underwater glider. Section 3 describes the CFD setup and hydrodynamic analysis. Section 4 introduces the Kriging-based optimization framework and algorithmic workflow. Section 5 discusses the optimization results and validation. Finally, Section 6 summarizes the main conclusions and outlines future research directions.

## 2. Ray-like Shape Parameterization Modeling

The shape parametric modeling of the ray-like underwater glider proposed in this paper is chiefly made up of three parts: the parametric modeling of the ray-like shape contour, the parametric modeling of the airfoil sections based on the CST method, and examples of shape parametric modeling and deformation. This section provides a detailed description of each part.

### 2.1. Ray-like Shape Contour Parameterization

**Figure 1:**
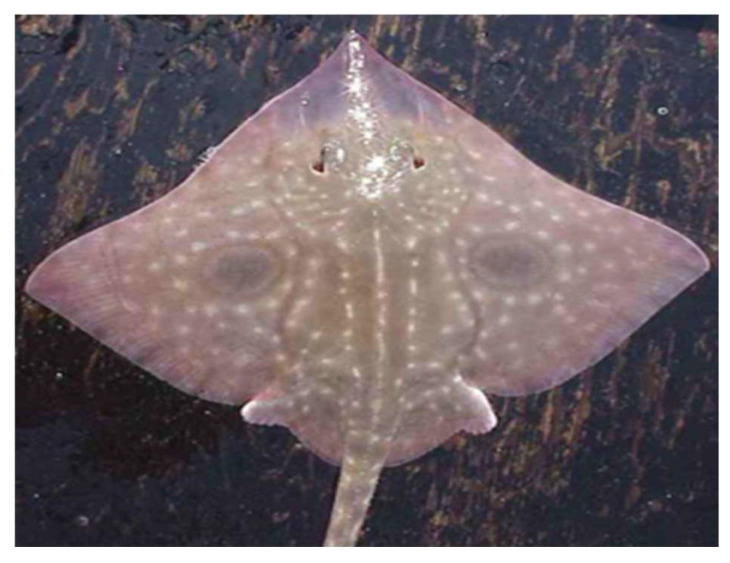
Picture of the manta ray fish shape.

Figure 1 shows the shape of the ray, whose unique wing-body integration, wide and flexible wing surface, and streamlined contour provide important inspiration for the bionic design of underwater gliders. This paper conducts the outline contour parameterization modeling of the underwater glider based on the natural form of the ray. First, referring to the shape characteristics of the ray, the overall geometric dimensions of the bionic glider are determined. The overall length is defined as the straight-line distance from the nose to the farthest wingtip, used to represent the longitudinal scale of the glider. The overall width is defined as the maximum span between the left and right wingtips, used to represent the spanwise scale of the glider. These two parameters serve as key constraints for the outline design of the underwater glider, directly affecting the structural layout, mass distribution, and hydrodynamic performance of the glider.

**Figure 2:**
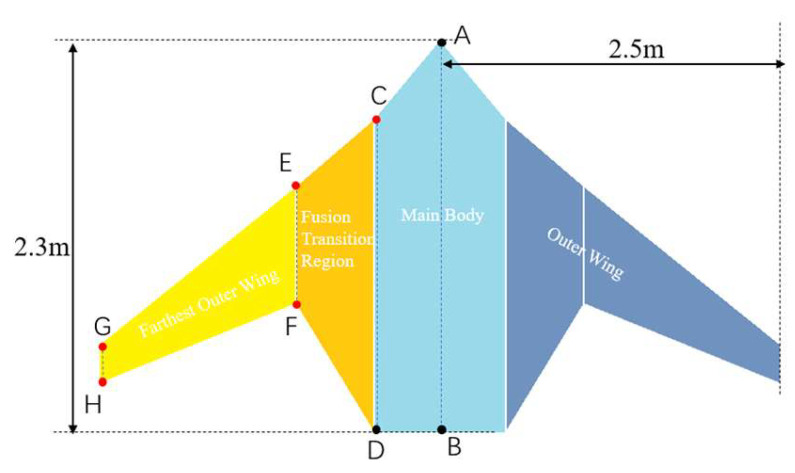
Schematic diagram of MRLUG shape outline parameters.

Based on the above dimensions, the overall contour diagram of the underwater glider is drawn, as shown in Figure 2. This contour diagram reflects the basic configuration characteristics of the biomimetic ray-like underwater glider and divides the shape into three main areas: the main body area, the fusion transition area, and the outer wing area.

The main body area primarily corresponds to the central fuselage section of the glider, which is the core region for bearing the main structure and primary mission payload of the platform. The outer shape of this area is mainly defined by the feature points A, B, C, and D in Figure 2. During the shape design process, its dimensions need to be comprehensively determined in conjunction with the internal load distribution and layout scheme. Once the internal load types and quantities are known, the maximum longitudinal projection position of this region’s contour is fixed. Therefore, in the optimization process, feature points A and B in Figure 2 are kept constant as boundary control points. Additionally, since the glider’s tail usually requires installation of dual propellers, the tail contour needs to maintain a certain linear structure to ensure the symmetric arrangement and efficient operation of the propellers. This simplifies the integration design and installation process of the propulsion system, so the corresponding feature point D is also set as a fixed parameter. In conclusion, during the shape optimization design, only feature point C is treated as the parameter for geometric deformation. By fine-tuning its position, optimization adjustments can be made to the fuselage curvature, hydrodynamic shape, and streamline transitions, thereby further improving the overall hydrodynamic performance of the glider while meeting internal spatial layout and system integration requirements.

The fusion transition area is located between the main body region and the outer wing region of the glider. It serves the function of connecting the central fuselage with the outwardly extending wing structure. The smoothness of this area directly affects the continuity and stability of the flow field and plays a key role in reducing fluid disturbances and improving hydrodynamic efficiency. The shape of this region is primarily defined by feature points C, D, E, and F in Figure 2. Points C and D are located near the main body region side of the transition zone and serve as the shared boundary between this region and the main body area. The design of these points is mainly carried out in the main body region. Points E and F are located on the side of the fusion transition area closer to the outer wing. The specific locations of these points significantly influence the curvature change, transition length, and spanwise profile of the shape. Therefore, in the shape optimization design, feature points E and F are set as the main geometric deformation parameters.

The outer wing region is located on both sides of the glider platform. It is a key structural unit responsible for generating lift, attitude control, and directional stability, and its shape design significantly influences the hydrodynamic performance of the entire platform. The profile of this region is defined by feature points E, F, G, and H in Figure 2. Points E and F are connected to the fusion transition area and are primarily determined through the shape design of the fusion transition region. Feature points G and H are located towards the wingtip, and their specific positions directly affect key geometric parameters such as the span, chord length variation, edge curvature of the outer wing and etc. Therefore, in the shape optimization design, feature points G and H are set as the primary parameters for geometric deformation. Table 1 lists the parametric variables of the ray-like glider’s outer shape and their corresponding functions.

### 2.2. CST-Based Airfoil Section Parametrization

A notable feature of the ray-like underwater glider is that each cross-section of its fuselage-wing blending configuration is composed of a specific airfoil section, designed to enhance hydrodynamic performance during underwater gliding. After completing the geometric parameterization of the overall shape, airfoil sections must also be parameterized for modeling.

This paper uses the CST (Class-Shape Transformation) parameterization method to model the airfoil sections [25] of the ray-like underwater glider. The CST method, advanced by Kulfan and Bussoletti [26], is a parameterization technique based on analytical expressions and has been widely applied in the parametric modeling and optimization design of airfoils, wings, and other aerodynamic shapes. The CST method is aimed at representing the shape geometry as a product of class functions and shape functions. By adjusting the control coefficients of the shape functions, the shape features can be flexibly controlled, enabling efficient parameterization. Compared to traditional shape parameterization and perturbation parameterization methods, the CST method offers significant advantages, including fewer parameters, higher modeling efficiency, better geometric smoothness, and stronger local control ability. These advantages make it highly suitable for optimization design of airfoils and fuselage-wing blended configurations.

For the parameterization of the 2D airfoil section of the ray-like underwater glider, the mathematical expression of the CST method can be written as:

(1) $$y\left(x\right)=C\left(x\right)\cdot S\left(x\right)+x\cdot \Delta y_{TE},$$

where C(x) is the class function, employed to define the fundamental shape of the geometric profile; S(x) is the shape function, which finely shapes the actual profile of the airfoil by a weighted combination of a series of smooth basis functions. For the symmetric airfoil used in this paper, the form of C(x) is:

(2) $$C\left(x\right)=x^{N_{1}}\cdot {\left(1-x\right)}^{N_{2}},$$

where x is the normalized chordwise coordinate, with a range of [0, 1]. $N_{1}$ determines the sharpness of the curvature at the leading edge, while $N_{2}$ controls the closure behavior of the trailing edge and the rate at which thickness converges.

S(x) is the shape function, which is typically represented using a weighted sum of *n*-th order Bernstein polynomials or B-spline basis functions. In the parameterization method used in this paper, the Bernstein polynomial is used to construct the shape function, which is expressed as follows:

(3) $$S\left(x\right)=\sum_{i=0}^{n}w_{i}[\frac{n!}{r!\left(n-r\right)!}x^{i}{\left(1-x\right)}^{n-i}],$$

where n represents the order of the polynomial, i is the exponent of the polynomial, and $w_{i}$ is the weight coefficient of the shape function. After the class function corresponding to the geometric shape is determined, the local features of the airfoil section could be finely managed by adjusting the values of $w_{i}$.

**Figure 3:**
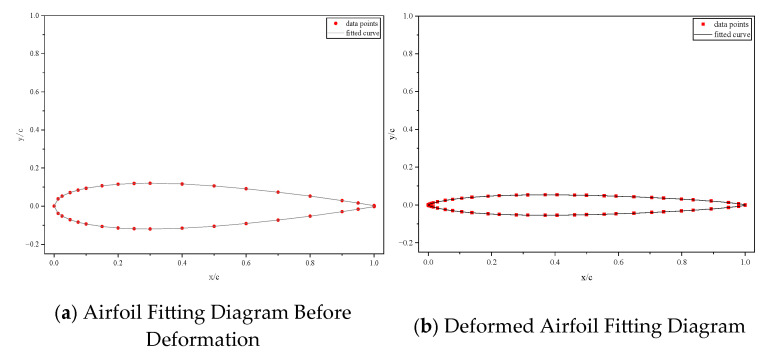
Airfoil Parametric Deformation Fitting Results.

This paper uses the classic NACA0012 symmetric airfoil as the initial geometric reference, and constructs a CST parameterization model with a fifth-order Bernstein polynomial to mathematically represent the airfoil section of the ray-like underwater glider. By introducing six key design variables ($N_{1},N_{2},w_{1},w_{2},w_{3},w_{4}$), the controllable deformation of the airfoil geometry is achieved under the strict curvature continuity constraint. This parameterization method retains the high-precision fitting capability of the CST model for complex curves while providing a flexible mathematical framework for the optimization design of bio-inspired airfoils through explicit control of the design variables, as shown in Figure 3.

### 2.3. Examples of Shape Parametric Modeling and Deformation

In short, the parametric modeling of the ray-like underwater glider’s shape is mainly achieved through contour parameters and airfoil parameters. The detailed introduction to the parametric variables is provided in Table 2 below.

**Figure 4:**
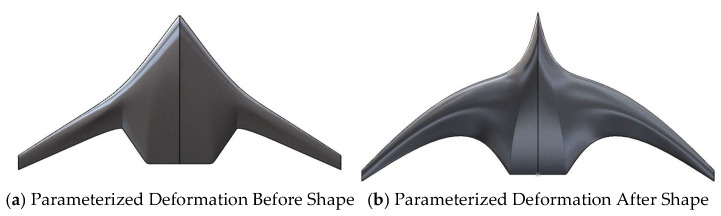
Comparison of the model before and after parameterized deformation.

Based on the aforementioned parametric variables, this paper creates a 3D model of the deformation using 3D modeling software, as shown in the figure below. The figure displays a portion of the model, where the airfoil sections are the data obtained after deformation through CST parametric modeling in Section 2.1, and the shape contour is deformed according to the parameters discussed in Section 2.2. Figure 4 presents a comparison between the models before and after parametric modeling.

## 3. CFD-Based Hydrodynamic Calculation and Flow Field Analysis

The lift–drag ratio of the ray-like underwater glider is the primary objective of its shape optimization design. To accurately assess the lift–drag ratio, this study utilizes Computational Fluid Dynamics (CFD) for precise hydrodynamic calculations. Additionally, flow field analysis is combined to identify key geometric regions that significantly influence the lift–drag ratio.

### 3.1. Mesh Division and Solver Setup for the Computational Domain

**Figure 5:**
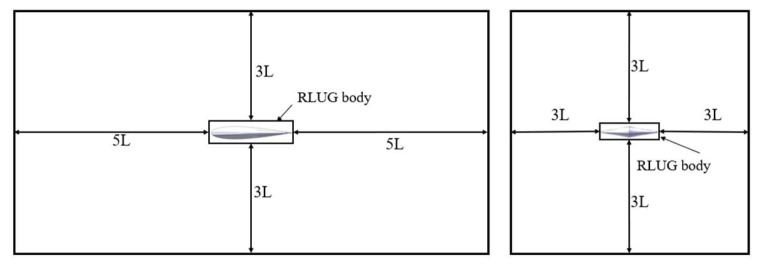
Schematic Diagram of the Main Dimensions of the Computational Domain.

The computational block is set as a rectangular shape, with dimensions of in the length, width, and height directions  [−5L, 5L] × [−3L, 3L] × [−3L, 3L], respectively (where L is the characteristic length). This setup helps prevent distortion of the flow field around the glider due to boundary conditions. The specific dimensions are shown in Figure 5 below.

**Figure 6:**
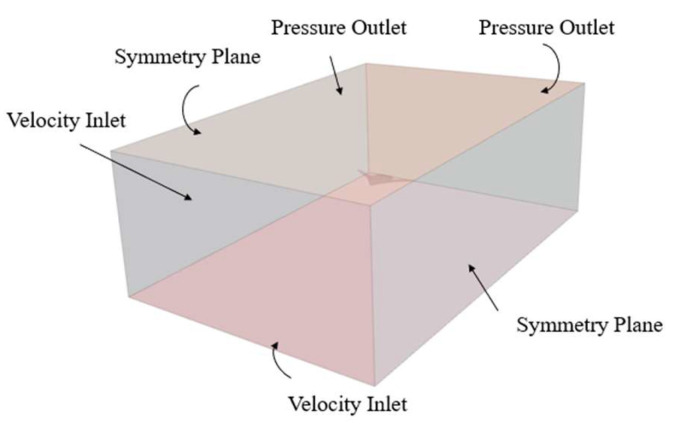
Schematic diagram of boundary conditions for the computational domain.

Considering factors which will affect the navigation of the Ray-Like Underwater Glider (RLUG) such as the attack angle, the boundary conditions are set as follows: the left and bottom surfaces of the rectangular computational block are set to velocity inlet conditions, the right and top surfaces are set to pressure outlet conditions, and the front and rear surfaces are set as symmetry boundary conditions. In addition, the surface of the RLUG is set as a no-slip wall condition. The specific setup is shown in Figure 6.

**Figure 7:**
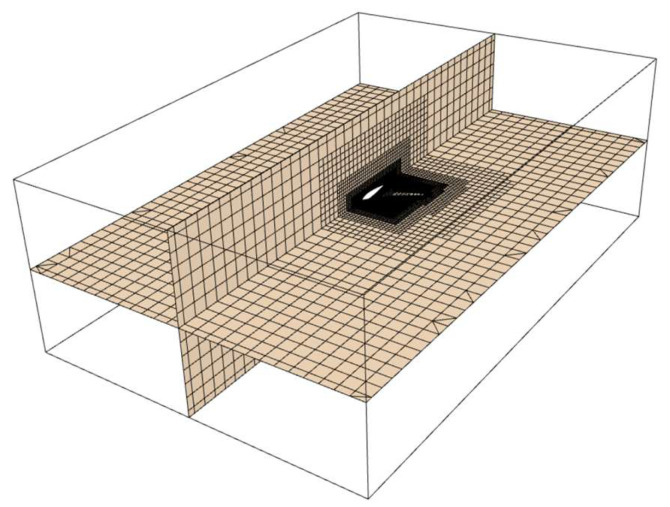
Mesh distribution of the Ray-like Underwater Glider (RLUG).

The Trimmed Cell Mesher and Prism Layer Mesher in STAR-CCM+ are employed to divide the computational domain into grids. The amount of prismatic layers is set to 9, with a volume growth rate of 1.2. Additionally, Local mesh refinement is applied to the RLUG surface. the mesh density around the object significantly affects the accuracy, while the computational domain far from the object has a negligible influence on the solution’s accuracy. Thus, to reduce the total mesh count while ensuring accuracy, the boundary layer prismatic mesh and surface reconstruction are disabled, and the boundary layer mesh size is adjusted to allow the mesh to gradually expand outward. The mesh distribution for the RLUG computational domain is exhibited in Figure 7.

The velocity inlet is set to ensure that the flow direction corresponds to the attack angle of the glider. The “SST k−ω” turbulence model is employed for the solution. The solver settings include: the maximum amount of iterations set to 1000 steps, and the convergence residual set to 1 × 10^−5^.

### 3.2. Experimental Validation

To ensure the accuracy of the CFD method used, this study first validates the reliability of the CFD approach by comparing simulation results with existing experimental data for the hydrofoil [27]. The experimental data, including the lift coefficient ($C_{L}$), drag coefficient ($C_{D}$), and flow field distribution, will serve as the baseline for preliminary validation. By calculating and comparing the lift-to-drag ratio (L/D) of the hydrofoil under the same conditions (e.g., 2 knots speed and 4° attack angle) with the experimental results, the CFD method is confirmed to accurately simulate the hydrodynamic performance, thus providing a reliable foundation for the subsequent simulation of the ray-like underwater glider (RLUG). Table 3 compares the results obtained from CFD calculations with the corresponding experimental data.

As can be seen from the Table 3 above, the maximum error between the CFD results and the hydrofoil experimental data is 4.8%, and it does not exceed 5%. Therefore, it can be concluded that the CFD results are reliable and accurate.

### 3.3. Mesh Independence Test

The quality and quantity of the mesh straightway affect the precision of the CFD results. In theory, increasing the number of mesh elements improves the computational accuracy. However, due to the limitations of computer processing power, an excessive number of mesh cells can significantly increase the computation time. Therefore, reducing the number of mesh elements while ensuring computational accuracy is a crucial issue that requires to be handled in the early stages of the computation. Mesh independence testing is a commonly used solution. By studying the computational accuracy under different mesh resolutions, it is possible to select a reasonable mesh density that balances both accuracy and computational efficiency.

For the sake of ensuring that the mesh satisfies both computational accuracy and an appropriate computation time, numerical simulations of the flow field around the Ray-like Underwater Glider (RLUG) were conducted using different mesh sizes. The calculations were based on the RANS equations and solved using the “SST k−ω” turbulence model. The lift–drag ratio was computed under a 2-knot speed and 4° attack angle condition. The specific results are shown in Table 4.

According to the table above, it can be observed that number of grids alters from 580,000 to 1.88 million, lift–drag ratio decreases from 8.50 to 7.37, indicating a significant change. However, when the grid is further refined to 3.32 million and 7 million, the lift–drag ratios are 7.32 and 7.34, respectively, and the variation becomes relatively stable. This suggests that the sensitivity of the calculation results to grid resolution has significantly decreased, and the numerical solution has converged. Considering both calculation accuracy and computational cost, a grid scheme with 1.88 million nodes was ultimately chosen for the subsequent simulation and analysis.

### 3.4. Flow Field Analysis of the Ray-like Underwater Glider

**Figure 8:**
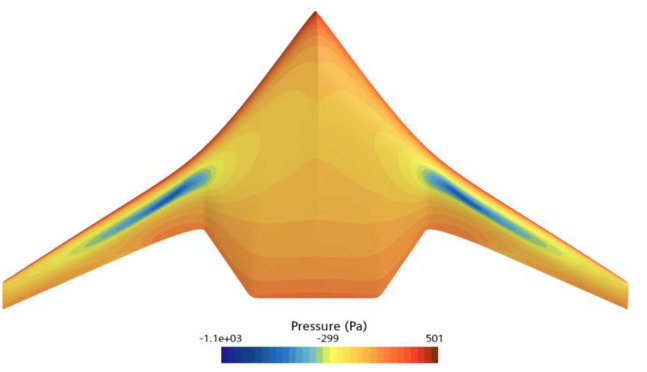
Pressure Contour Plot on the Bottom Surface of RLUG.

**Figure 9:**
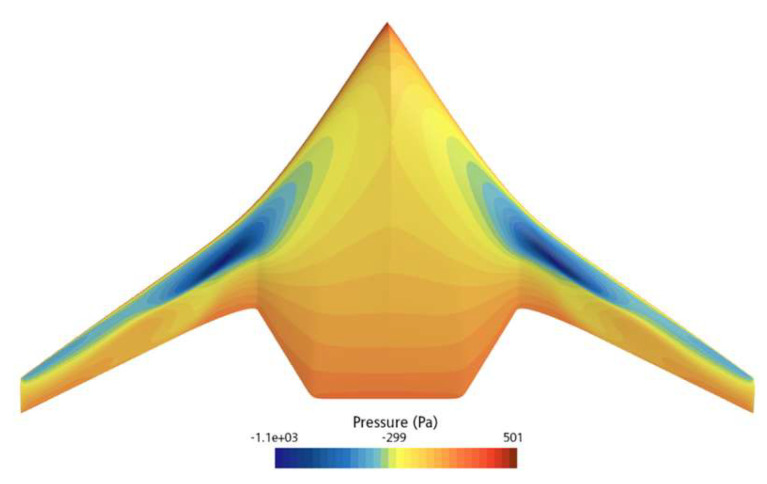
Pressure Contour Plot on the Top Surface of RLUG.

Figure 8 and Figure 9 show the pressure distribution contour plots of the underwater glider at a speed of 2 knots and an attack angle of 4°. Figure 8 illustrates the pressure distribution on the bottom surface of the RLUG, while Figure 9 shows the pressure distribution on the top surface. From the figures, it is evident that, under the condition of a 4° attack angle, the incoming flow significantly impacts the abdominal region of the glider, resulting in higher dynamic pressure, particularly in the front edge area, which forms a notable high-pressure zone on the lower surface. In contrast, the dorsal region, due to the relatively negative pressure effect, exhibits a smaller pressure gradient. The pressure difference between the top and bottom surfaces is the primary source of lift for the glider.

**Figure 10:**
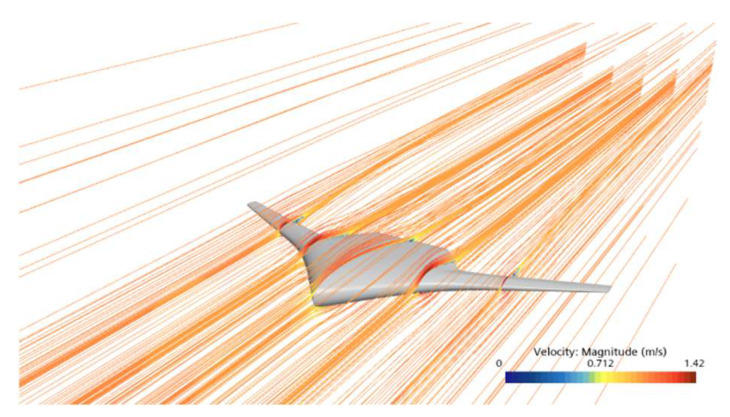
Streamline distribution around the RLUG body.

Figure 10 presents the streamline distribution of the RLUG under a flow velocity of 2 knots and an angle of attack of 4°, which is used to further analyze the flow field structure around the glider. The simulation results show that the incoming flow impinges on the front surface of the glider at a certain angle of incidence. Due to viscous effects, an attached boundary layer forms along the body surface and gradually extends toward the tail as the body curvature changes. As the flow develops downstream toward the tail region, it is subjected to an adverse pressure gradient near the trailing edge, leading to a gradual decrease in fluid kinetic energy. Consequently, the boundary layer thickness increases progressively, and localized flow separation eventually occurs near the trailing edge.

According to the above, it can be concluded that the glider exhibits excellent overall aerodynamic performance under small attack angle conditions, with the incoming flow generally flowing smoothly along the body surface. However, the leading edge region experiences stronger impact pressure due to direct flow impingement, while the tail region is affected by adverse pressure gradients, which poses a potential risk of boundary layer separation. Therefore, in subsequent shape optimization design, particular focus should be paid to the streamlining and influence resistance of the leading edge region, as well as the control of adverse pressure gradients and strategies for separation suppression in the tail region, in order to achieve better hydrodynamic performance.

## 4. Sequential Optimization Framework Based on the Kriging Model

**Figure 11:**
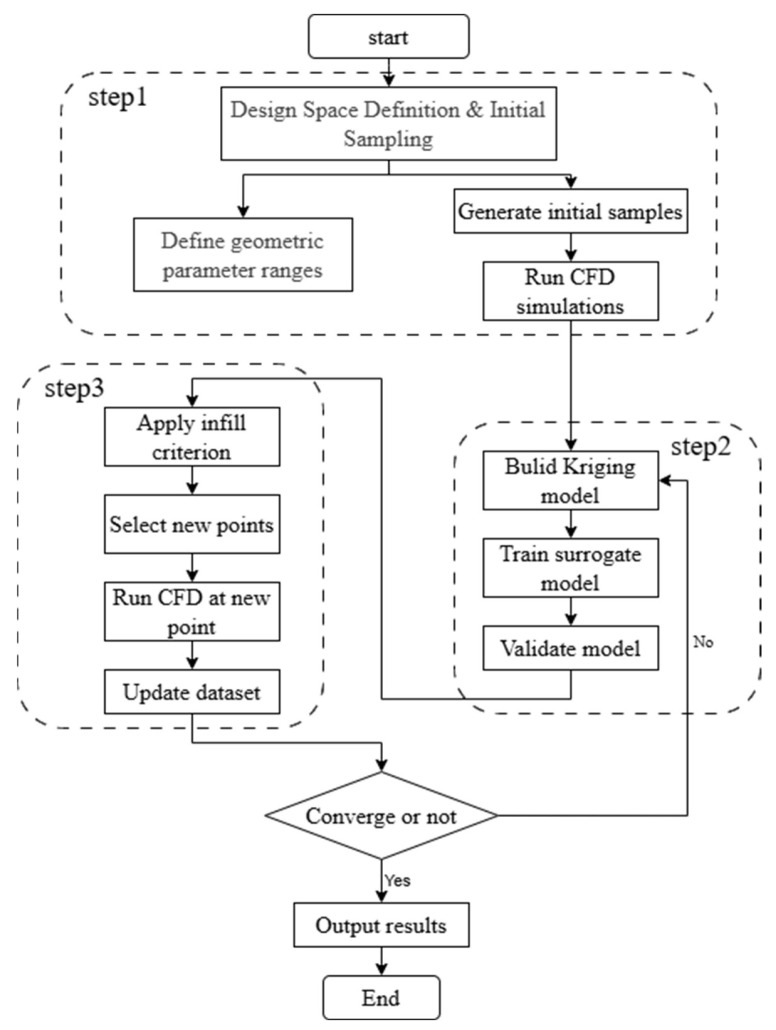
Sequential Optimization Flowchart Based on Kriging Model.

In this shape optimization design of a ray-like underwater glider, due to the involvement of multiple geometric parameters, conventional optimization algorithms (such as genetic algorithms, particle swarm optimization, etc. [28,29]) require thousands of CFD simulations, resulting in high computational costs and low search efficiency. To improve optimization efficiency and reduce CFD calls, this article constructs a sequential optimization framework based on the Kriging model. By dynamically adding sample points in the design space and iteratively updating the surrogate model, the efficient optimization of the ray-like underwater glider’s shape is achieved. The process is demonstrated in Figure 11. The optimization process mainly includes the following four steps: design space definition and sample collection, Kriging model construction, sampling criteria design and sample update, and optimization convergence criterion setting. The following sections will provide detailed explanations of each step.

- Step 1: Design Space Definition and Sample Collection

First, based on the geometric structural characteristics of the glider, key design variables to be optimized (such as the coordinates of feature points C,E,F,G, and H, as well as key control parameters for the airfoil sections) and their upper and lower limits are determined. A design space with multiple variable dimensions is then constructed. To ensure the surrogate model has good prediction accuracy in the initial stage, the Latin Hypercube Sampling (LHS) [30] method is used to evenly generate the initial sample points within this space. Each point represents a combination of geometric parameters, and the corresponding lift–drag ratio response is obtained through numerical simulation. These input-output sample pairs form the data foundation for the Kriging model.

- Step 2: Kriging Model Construction

To avoid frequent calls to the high-cost CFD solver, this study constructs a Kriging surrogate model [31] for the lift–drag ratio of the ray-like underwater glider. This surrogate model is used to efficiently predict the performance response for any combination of geometric parameters during the optimization process.

The Kriging model is a statistical interpolation modeling method. Its fundamental idea is to treat the unknown function as consisting of a deterministic trend term and a Gaussian process term with zero mean, as follows:

(4) y(x)=μ+Z(x),

where μ is the global trend model (usually set as a constant), and Z(x) is a stationary random process with a zero mean and variance of $\sigma^{2}$. Its covariance function is defined as:

(5) $$Cov\left[Z\left(x_{i}\right),Z\left(x_{j}\right)\right]=\sigma^{2}R\left(x_{i},x_{j}\right),$$

where $R\left(x_{i},x_{j}\right)$ is the correlation function, which reflects the spatial correlation between sample points. Typically, Gaussian, exponential, or Matérn correlation functions are used, and they satisfy the condition that the correlation is maximized when the distance between samples is zero, and decreases gradually toward zero with the distance increasing.

Based on the known *n* sample points $\left\{x_{1},\ldots ,x_{n}\right\}$ and their corresponding response values $y_{i}$, the predicted value of the Kriging model can be expressed as a weighted linear combination:

(6) $$\hat{y}\left(x\right)=\sum_{i=1}^{n}\lambda_{i}\left(x\right)y_{i},$$

To obtain the weighting coefficients $\lambda_{i}$, the Kriging model minimizes the predicted mean square error while satisfying the unbiased condition, thus forming the following constrained optimization problem:

(7) $$Min.\;\;\;\;\;\;\;\;\;E\left[{\left(\hat{y}\left(x\right)-y\left(x\right)\right)}^{2}\right],\;\;s.t.\;\;\;\;\;\;\;\;\;\sum_{i=1}^{n}\lambda_{i}=1,\;$$

By introducing the Lagrange multiplier method, the Lagrangian function is constructed as follows:

(8) $$L=\sigma^{2}\left(\lambda^{T}R\lambda -2\lambda^{T}r+1\right)+2\eta \left(\lambda^{T}\cdot 1-1\right),$$

where λ is an n-dimensional column vector, with elements representing the corresponding weights for the sample points when predicting a new point; R is an n × n correlation matrix, with elements representing the correlation function values between sample points; r is the correlation function value vector between the prediction point and the sample points; and 1 is an n-dimensional column vector.

After solving, the final Kriging prediction model can be written as:

(9) $$\hat{y}\left(x\right)=\mu +r^{T}R^{-1}\left(y-\mu \cdot 1\right),$$

Additionally, the Kriging model can also estimate the mean square error (MSE) at the prediction point, which is expressed as:

(10) $$MSE\left(x\right)=\sigma^{2}\left[1-r^{T}R^{-1}r+\frac{{\left(1-1^{T}R^{-1}r\right)}^{2}}{1^{T}R^{-1}1}\right],$$

- Step 3: Point Addition Criterion Design and Sample Update

To continuously improve the prediction accuracy of this Kriging surrogate model and gradually approach the global optimal solution in the design space, this paper introduces a sequential sampling strategy based on the point addition criterion [32]. By dynamically adding new sample points, this surrogate model is continuously updated and refined. During the sequential point addition process, the selection of sample points not only affects the model’s approximation ability but also plays a great role in the accuracy and convergence efficiency of the final optimization result.

The commonly used Mean Squared Prediction (MSP) criterion focuses on the global prediction uncertainty of the surrogate model, tending to add new points in regions where the model error is large. It tends to add new points in regions with large model errors, thereby improving the model’s fitting accuracy across the entire design space and avoiding underfitting in certain areas. On the other hand, the Expected Improvement (EI) criterion emphasizes the local improvement potential in the optimization direction, prioritizing sampling in regions where a significant improvement in the objective function is likely, which results in good local search performance. Both criteria have their advantages but also limitations: using only MSP may lead to an evenly distributed sampling pattern without directionality, making it difficult to converge quickly to the optimal solution. Meanwhile, relying solely on EI may result in getting trapped in local optima, ignoring the overall predictive accuracy of the model.

Therefore, to enhance the overall reliability of the Kriging model and improve the stability and convergence efficiency of the optimization process, this paper proposes an effective combination of the MSP and EI criteria. In each sampling round, both the model’s uncertainty and the potential for improvement near the optimal value are considered. On the one hand, MSP is employed to maintain the global prediction accuracy of the surrogate model, while EI is leveraged to guide the optimization toward better designs, balancing global exploration and local refinement. For the ray-like underwater glider, the specific mathematical model descriptions of the MSP and EI sampling criteria are as follows:

MSP Sampling Criterion:

The MSP is aimed at directly searching for the position in the design space where the predicted value of the objective function is the smallest based on the current surrogate model’s predictions, as the new sample point, i.e.,:

(11) $$x_{new}=\arg\min\hat{y}\left(x\right),\;\;x\in D,$$

where D is the design variable space, and $\hat{y}\left(x\right)$ is the predicted minimum value of the objective function.

For the manta ray-inspired underwater glider, the MSP sampling criterion adds sample points by solving the following constrained sub-optimization problem:

(12) $$\begin{array}{c} Min.\;\;\;\;\;\;\;\;\;\;\;\;\;\;\;\hat{y}\left(x\right),\;\;\;\;\;\;\;\;\;\;\;\;\;\;\;\;\\ s.t.\;\;\;\;\;\;\;\;\;\;\;\;\;\;\;x_{l}\leq x\leq x_{u}, \end{array}$$

In the equation, $\hat{y}(x)$ represents the surrogate model of the objective function, and $g_{i}$ refers to the geometric constraints. This problem can be solved using a multi-start Sequential Quadratic Programming (SQP) optimization method.

EI Sampling Criterion:

EI is an advanced sampling strategy that combines the predicted values of the surrogate model and its uncertainty. Let the current optimal objective function value be $y^{*}$, and the Kriging model predicts a normal distribution at point x, with a mean value of $\hat{y}(x)$ and a standard deviation of σ(x). The expected improvement of the objective function $I\left(x\right)=max(0,y^{*}-\hat{y}(x))$ is given by:

(13) $$E\left[I\left(x\right)\right]=\left[y^{*}-\hat{y}\left(x\right)\right]\Phi \left(Z\right)+\sigma \left(x\right)\phi \left(Z\right),$$

where $Z=\frac{y^{*}-\hat{y}\left(x\right)}{\sigma (x)}$, Φ(Z) and ϕ(Z) are the cumulative distribution function (CDF) and probability density function (PDF) of the standard normal distribution, respectively.

For the ray-like underwater glider, the sample points are added by solving the following constrained sub-optimization problem to maximize the EI value.

(14) $$\begin{array}{c} Max.\;\;\;\;\;\;\;\;\;E\left[I\left(x\right)\right],\;\;\;\;\;\;\;\;\;\;\\ s.t.\;\;\;\;\;\;\;\;x_{l}\leq x\leq x_{u}, \end{array}$$

- Step 4: Convergence Criterion

In this paper, three convergence criteria are set for the optimization of the ray-like underwater glider’s shape: If the difference between the four continuous sample points is less than 1%, the optimization process will be terminated, and the current optimal parameter set will be output as the final solution, aiming to avoid unnecessary iterations. The criterion for determining convergence is described as follows:

$$\delta_{i}=\frac{y_{k}-y_{k-1}}{y_{k-1}}<1\%,$$

where $y_{k}$ is the lift–drag ratio obtained from the k-th interion, $\delta_{i}$ means the difference between the continuous iterations. The iteration is considered optimal when the deviation $\delta_{i}$ remains below 1% for four continuous steps.

To validate the stability of the proposed optimization framework with respect to initial random sampling, this study further selects two standard test functions with known global optima: Sphere and Hartmann [33,34]. These functions are used to construct optimization scenarios with low and medium dimensions, in order to comprehensively assess the global optimization capability of the proposed framework.

In each case, initial samples are first randomly generated within the given design space using LHS. The optimization process is then carried out using the Kriging-based Sequential Optimization Framework for iterative optimization. Finally, each setup is independently run 30 times (with different seeds), and the mean and variance of the optimal objective function values are recorded, as shown in Table 5, to evaluate the impact of random initialization on the optimization results.

Taken together, the above results demonstrate that the Kriging-based sequential optimization framework proposed in this study exhibits strong convergence performance and stability across different types of test functions. For the Sphere function, the method achieves solutions extremely close to the theoretical optimum with minimal variance, indicating excellent fundamental convergence capability. In the multimodal Hartmann function, the algorithm consistently obtains results near the optimum, reflecting its strong global search ability. Overall, the proposed optimization approach demonstrates clear advantages in accuracy, stability, and global exploration capability.

## 5. Analysis of the Shape Optimization Results for the Ray-like Underwater Glider

The shape optimization design of a ray-like underwater glider is carried out using the previously proposed shape parametric method, hydrodynamic calculation method, and the Kriging sequential optimization framework. The effectiveness of the proposed method is verified by a comparative analysis of the obtained optimization results.

### 5.1. Optimization Problem Description

**Figure 12:**
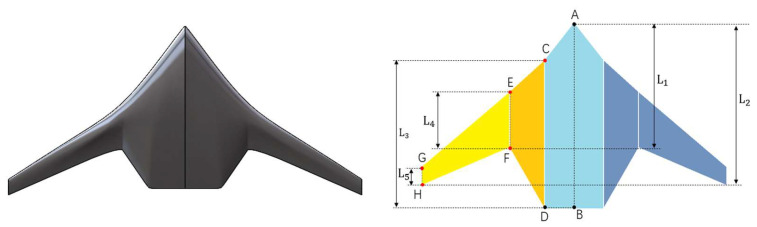
Geometric parameters of the initial prototype model for optimization.

To effectively validate the practical effectiveness of the shape parameterization modeling and optimization design method proposed in this study, the design of a prototype, as shown in Figure 12, was selected as the specific research object for the shape optimization.

In shape parameterization of the manta-ray-inspired underwater glider, to prevent the intersection of the leading-edge feature points C,E,G and trailing-edge feature points F,H, the parameterization of feature points C,E,F,G and H is transformed into five core shape parameters ($L_{1},L_{2},L_{3},L_{4},L_{5}$). The specific definitions are as follows: The lateral position of feature point F remains unchanged, and its longitudinal position is used as an adjustable parameter, denoted as $L_{1}$. In the same way, the lateral position of feature point H remains constant, and the longitudinal position is an adjustable parameter, denoted as $L_{2}$. The lateral position of feature point C coincides with feature point D, and its longitudinal position is precisely controlled by the chord length $L_{3}$ of the *CD* segment. The lateral position of feature point E coincides with feature point F, and the longitudinal position of feature point E can be adjusted by the chord length $L_{4}$ of the EF segment. The lateral position of feature point G coincides with feature point H. After determining the longitudinal position of point H ($L_{2}$), the longitudinal position of feature point G can be adjusted by the chord length $L_{5}$ of the *GH* segment.

In the parameterization of the manta-ray-inspired underwater glider’s airfoil profile, to minimize the deviation between the CST parameterization method’s fitted curve and the original data points, a 5th-order Bernstein polynomial is used to construct the parameterization model. In this model, $N_{1}$ is used to change the leading-edge curvature radius, controlling the sharpness of the airfoil’s leading edge, and $N_{2}$ controls the degree of closure of the trailing edge. The $w_{i}$ are the weight coefficients of the Bernstein polynomial terms in the shape function, allowing precise control of the airfoil profile’s local characteristics.

In terms of optimization objectives, this paper aims to maximize the lift–drag ratio of the glider through shape optimization design, thereby enhancing its underwater gliding efficiency and range performance. For the selection of optimization design variables, a comprehensive consideration of shape flexibility, modeling efficiency, and lift–drag ratio influencing factors was made. As a result, 12 optimization variables were chosen, including the longitudinal coordinates of two shape feature points ($L_{1},L_{2}$), the chord lengths of three key regions ($L_{3},L_{4},L_{5}$), six airfoil profile parameters ($N_{1},N_{2},w_{1},w_{2},w_{3},w_{4}$), and the attack angle α. The specific parameters and their meanings are shown in the Table 6 below.

In addition, during the optimization process, reasonable variation ranges for each design variable are set. On one hand, these ranges provide the optimization algorithm with sufficient search space to fully explore the potential for superior shapes. On the other hand, they also help avoid issues such as a sharp increase in computational cost and poor convergence due to an excessively large design space. Specifically, the variation range for each geometric design parameter and airfoil section control parameter is set to be between 0.8 and 1.2 times its initial value, while the attack angle parameter variation range is set to [0, 8] degrees.

In summary, this paper establishes the mathematical model for this shape optimization of the manta ray-inspired underwater glider, and the specific expression is shown as follows:

(15) $$\begin{array}{cc} \mathrm{Max}. & L/D,\\ W.r.t. & L_{i},N_{j},,w_{k},\alpha ,\\ s.t. & 0.8\leq L_{i}/L_{i0}\leq 1.2,i=1,\ldots 5,\\ & 0.8\leq N_{j}/N_{j0}\leq 1.2,k=1,2,\\ & 0.8\leq w_{k}/w_{k0}\leq 1.2,k=1,2,3,4,\\ & 0\leq \alpha \leq 8, \end{array}$$

where, L/D represents the lift–drag ratio of the underwater glider, $L_{i}$ denotes the parameter values after the transformation of the five shape contour feature points, $L_{i0}$ represents the initial parameter values of the transformed design variables of the shape contour, $N_{j}$ refers to the parameters of the leading and trailing edges of the airfoil section, $N_{j0}$ represents the initial parameter values of the leading and trailing edge control variables, and $w_{k}$ denotes the weight coefficients of the shape function, with $w_{k0}$ being the initial weight coefficients of the shape function.

### 5.2. Optimization Results Analysis

In the process of optimization and hydrodynamic numerical simulations, the computational platform used in this study is the Intel i9-13900K processor. This processor has a base clock speed of 3.0GHz and is equipped with 24 cores and 32 threads, providing robust computational performance.

**Figure 13:**
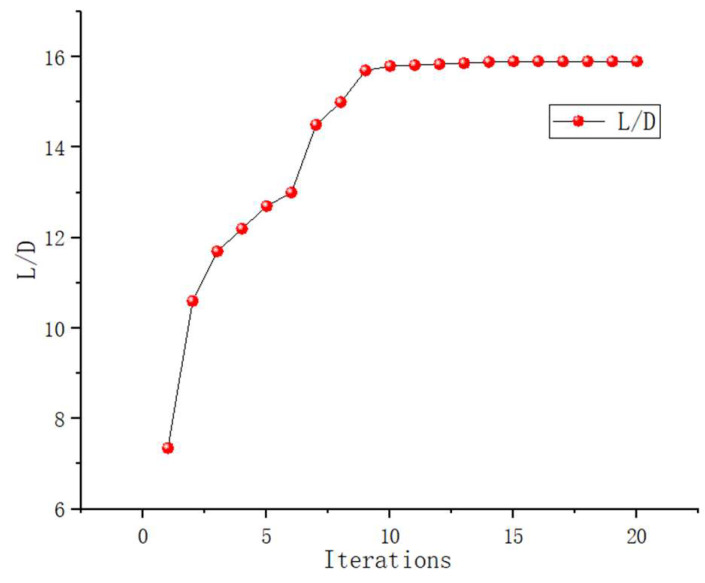
Convergence History of RLUG Shape Optimization.

The optimization problem (15) is solved by using the proposed method. First, 70 sample points are selected by the LHS method as initial sample points, and calculations are performed at each sample point to obtain the corresponding L/D. These results serve as the initial sample set for the subsequent Kriging surrogate model. Next, a Kriging model for the L/D ratio is established. Finally, the sub-optimization problems (14) and (12) are solved by PSO optimization method, with sample points being dynamically added until the optimization converges. The convergence condition is set to the difference between the four continuous sample points being less than 1%. Figure 13 below illustrates the convergence of the entire optimization process.

Figure 12 shows the optimization converged after 20 iterations with a total computation time of approximately 114 h and the optimized lift–drag ratio (L/D) reaches 15.9, which represents an improvement of approximately 116% compared to the initial design value of 7.35

In each iteration, sample points were added in parallel using the MSP and EI methods, resulting in a total of 20 iterations and 33 additional sample points. Table 6 presents the error between the CFD values and the Kriging predicted values at each iteration when new sample points were added. As shown in Table 7, with the increasing number of sample points, the model was progressively updated. The error between the predicted values of the Kriging surrogate model and the actual CFD values gradually decreased, and at the optimal point, it progressively approached the true CFD results.

**Figure 14:**
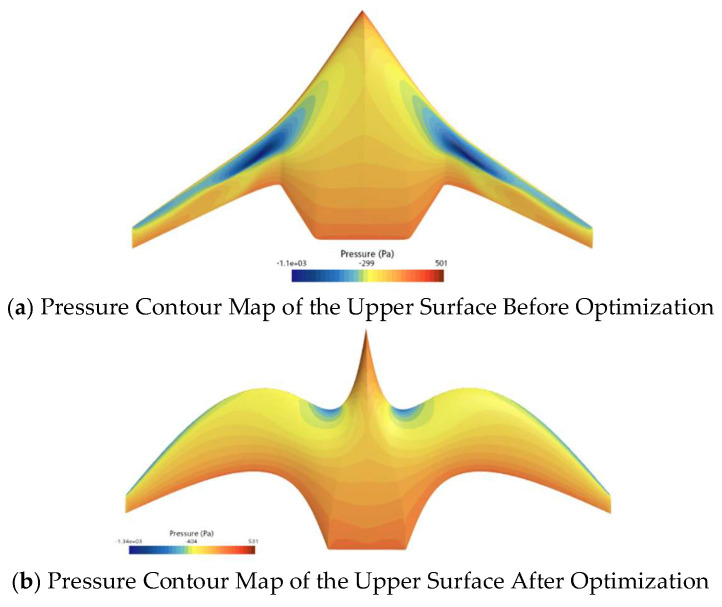
Comparison of Pressure Distribution on the Upper Surface Before and After Optimization.

**Figure 15:**
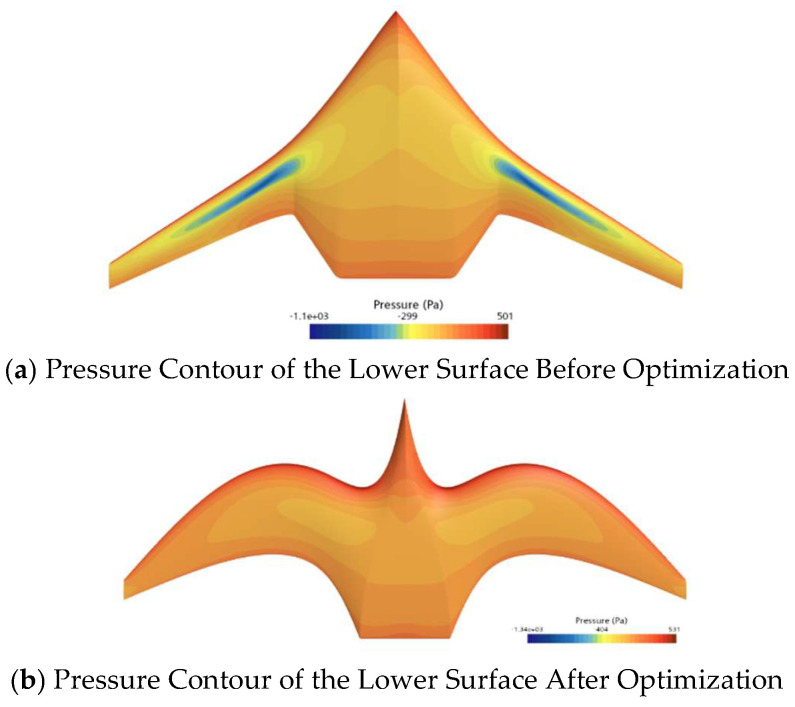
Comparison of Pressure Distribution on the Lower Surface Before and After Optimization.

Figure 14 compares the pressure distribution on the upper surface of the initial and optimized shapes of the ray-like underwater glider, while Figure 15 compares the pressure distribution on the lower surface of the initial and optimized shapes. As shown in Figure 14, the low-pressure area on the upper surface of the optimized shape is smaller than that of the initial shape, especially noticeable at the leading edge. From Figure 15, it can be seen that the high-pressure area on the lower surface of the optimized shape is larger than that of the initial shape. Overall, considering the pressure distributions on both the upper and lower surfaces, the optimized shape generates greater lift than the initial shape, thus improving the lift–drag ratio.

### 5.3. Analysis of Optimization Efficiency

To validate the efficiency of the sequential optimization method based on the Kriging model proposed in this study, a comparative analysis with the traditional optimization method NSGA was performed. Given that the optimization involves 12 variables, the traditional NSGA method requires thousands of CFD calculations to obtain the optimal solution, resulting in significant computational cost. Therefore, in this study, the NSGA optimization algorithm was stopped after 103 CFD evaluations, and the optimization results obtained at this point were compared with those from the proposed optimization method. Table 8 compares the optimization results obtained by the NSGA method and the proposed Kriging-based sequential optimization method after a total of 103 CFD evaluations on the same computational platform of the Intel i9-13900K processor.

As shown in Table 7, under the same conditions—optimizing 12 variables and performing 103 CFD evaluations—the NSGA method achieved a lift-to-drag ratio of 9.5, whereas the sequential optimization method based on the Kriging surrogate model proposed in this study resulted in a lift-to-drag ratio of 15.9, which is an improvement of 67.3% over the NSGA method. This demonstrates that the sequential optimization method based on the Kriging surrogate model is indeed more efficient than the traditional NSGA optimization method.

## 6. Conclusions

This paper proposes an efficient and flexible parametric modeling and optimization design method for the shape design of an underwater glider inspired by rays. Firstly, by extracting key feature points of the manta ray shape, a parametric model for the glider’s external profile was established, providing a clear geometric control mechanism for shape adjustment. On this basis, the Class-Shape Transformation (CST) method is applied to parametrize the airfoil cross-section, enabling high degrees of freedom in modeling both local and overall features of the glider shape. Based on the CFD method, the hydrodynamic performance of the underwater glider is computed, obtaining highly accurate predictions for key hydrodynamic parameters such as lift and drag. To improve optimization efficiency, a shape optimization design framework based on the Kriging surrogate model is developed, introducing a dynamic sampling strategy. By solving the sub-optimization problems of maximizing EI and minimizing MSP criteria, high-value sample points were dynamically added, significantly improving the convergence speed and global optimization capability of the process.

In addition, to confirm the effectiveness of the proposed shape parametric method and optimization design framework, a case study on the shape optimization of a ray-like underwater glider was conducted. The optimization results indicate that, compared to the initial shape, the optimized underwater glider exhibits more uniform surface pressure distribution, effectively reducing local high-pressure regions and improving its fluid dynamics characteristics. Meanwhile, the lift–drag ratio of the glider increased by 116%, significantly enhancing its hydrodynamic performance. These results provide strong evidence for the effectiveness and practical value of the shape parametric method and optimization framework in the shape optimization of ray-like underwater gliders. They also highlight the broad application potential of this method in the design and performance enhancement of underwater vehicles.

## Figures and Tables

**Table 1:** Shape Outline Parameter Table.

Title 1	Title 2
C	The longitudinal position of feature point C, which is the curvature control point at the tail of the main body region, is optimized through positional adjustments to fine-tune the body contour curvature and improve fluid transition performance.
E	The longitudinal position of feature point E, which marks the starting point of the outer wing in the fusion transition zone, controls the spanwise contour shape of the transition zone and the continuity of the flow field.
F	The longitudinal position of feature point F, which marks the termination point of the outer wing in the fusion transition zone, determines the length of the transition zone and is a key parameter for fluid stability.
G	The longitudinal position of feature point G, which is the leading edge control point of the outer wing, governs the wing span, chord length, and the design of the sweep angle.
H	The longitudinal position of feature point H, which is the trailing edge control point of the outer wing, controls the wing edge curvature and is a core parameter for heading stability.

**Table 2:** Parametric Variables of the Ray-like Underwater Glider Shape.

Title 1	Title 2
C,E,F,G,H	These five feature points, together with points *A, B,* and *D*, form the set of shape characteristic points for the ray-like underwater glider, which determines the overall shape of the glider.
$N_{1}$	Change the radius of curvature at the leading edge of the airfoil section.
$N_{2}$	Change the closure behavior of the trailing edge of the airfoil section.
$w_{i}$	Finely control the local characteristics of the airfoil section.

**Table 3:** The comparison between the experimental data and CFD results.

Attack Angle (Degree)	Lift–Drag Ratio by CFD	Lift–Drag Ratio by Experiment	Error (%)
2	18.4	18.73	−1.8%
4	22.6	23.76	−4.8%
6	24.7	24.15	2.3%

**Table 4:** Lift–drag ratio Simulation Results for Different Mesh Sizes.

Mesh Node Count (10,000s)	Lift–Drag Ratio (L/D)
58	8.50
94	7.70
188	7.37
332	7.32
700	7.34

**Table 5:** Test Results of Kriging-based Sequential Optimization Framework.

Test Functions	Theoretical Optimal Value	Statistical Mean of the Optimal Values	Variance of the Optimal Values
Sphere	0	0.022	0.012
Hartmann	0	0.071	0.025

**Table 6:** Optimization Variables and Their Meanings.

Parameter Symbols	Meaning
$L_{1}$	Longitudinal position of feature point F
$L_{2}$	Longitudinal position of feature point H
$L_{3}$	The chord length of segment CDdecides the location of feature point D.
$L_{4}$	The chord length of segment EF determines the location of feature point E
$L_{5}$	The chord length of segment GHdetermines the location of feature point G.
$N_{1}$	The sharpness of the airfoil leading edge.
$N_{2}$	The degree of closure of the airfoil trailing edge.
$w_{i}$	The weight coefficients of the shape function at each order.
α	Attack angle

**Table 7:** Validation results of the Kriging model.

Iterations	CFD Evaluations per Round	The Validation Accuracy of the Kriging Model		The Number of Added Sample Points
		MSP-Based Sampling	EI-Based Sampling	
1	1	8.35%	8.53%	2
2	2	7.89%	7.93%	2
3	2	7.64%	7.55%	2
4	2	7.32%	7.43%	2
5	2	7.15%	7.22	2
6	1	7.04%	——	1
7	2	6.95%	6.86%	2
8	2	6.87%	6.75%	2
9	2	6.68%	6.63%	2
10	2	6.56%	6.52%	2
11	2	6.42%	6.31%	2
12	2	6.37%	6.25%	2
13	2	6.13%	6.06%	2
14	1	5.85%	5.88%	2
15	1	5.56%	——	1
16	2	5.29%	5.30%	2
17	2	5.10%	4.77%	1
18	2	5.06%	——	1
19	1	5.02%	——	1
20	1	4.98%	——	0

**Table 8:** Comparison of efficiency between NSGA and Sequential Optimization based on Kriging.

Comparison Items	NSGA	Sequential Optimization Based on Kriging
Number of variables	12	12
CFD simulation time (Total time of 103 times)	114 h	114 h
optimized result	9.5	15.9

## Data Availability

This study did not generate or analyze any new datasets. All information supporting the findings of this work is contained within the article. Data sharing is not applicable as the research does not involve biological samples, human data, or any ethical issues.
